# Exacerbation and severity of allergic symptoms during pregnancy and their impact on mental health

**DOI:** 10.1097/JW9.0000000000000002

**Published:** 2022-03-22

**Authors:** Kaori Yonezawa, Megumi Haruna, Kiwako Yamamoto-Hanada, Yukihiro Ohya

**Affiliations:** a Department of Midwifery and Women’s Health, Division of Health Sciences and Nursing, Graduate School of Medicine, The University of Tokyo, Tokyo, Japan; b Department of Health Quality and Outcome Research, Division of Nursing Systems, Global Nursing Research Center, Graduate School of Medicine, The University of Tokyo, Tokyo, Japan; c Allergy Center, National Center for Child Health and Development, Tokyo, Japan

**Keywords:** Allergic rhinitis, atopic dermatitis, depression, disease exacerbation, pregnant women

## Abstract

Pregnant women with allergies face difficulties, as they tend to avoid medication for symptom control during pregnancy. Moreover, some women experience exacerbations during this period. These difficulties can affect their mental health.

**Objective:**

This study aimed to investigate the factors influencing allergy exacerbations during pregnancy and examine the effects of allergy symptoms on the mental health of pregnant women.

**Methods:**

A cross-sectional study was conducted through web-based research in March 2020, amid the COVID-19 pandemic. Pregnant women were collected from 3 groups (target: 150 participants in each group): atopic dermatitis (AD), allergic rhinitis, and without allergies. We evaluated mental health using Kessler Psychological Distress Scale (K6) and health-related quality of life using the Short Form-8 questionnaire.

**Results:**

Overall, 202 (49.1%) of 411 pregnant women had depressive symptoms, and 45 (10.9%) had severe depressive symptoms. Women with allergies had significantly worse mental health than those without allergies. Among 119 AD patients, 36 (30.3%) experienced exacerbations during pregnancy. Of them, 11 (30.6%) did not consult a practitioner and endured the exacerbation. A significant association was observed between severe prepregnancy symptoms, job situation, coping with symptoms of AD during pregnancy, and AD exacerbation. Among 210 rhinitis patients, 17.1% experienced rhinitis exacerbation during pregnancy. The presence of rhinitis symptoms in winter and the second trimester was significantly associated with exacerbation.

**Conclusion:**

This study revealed higher rates of depressive symptoms among pregnant women with allergies. The psychological well-being of pregnant women with AD or allergic rhinitis should be considered.

**What is known about this subject in regard to women and their families?**

- Pregnant women with allergies face difficulties, as they tend to avoid medication for symptom control during pregnancy.
- Pregnant women with allergies (defined as positive immunoglobulin E sensitization) are at risk of depression and lower quality of life (QoL).
- Some women experience exacerbations during this period.

**What is new from this article as messages for women and their families?**

- Pregnant women with AD and allergic rhinitis experienced an exacerbation of allergic symptoms during pregnancy in 30.3% and 17.1%, respectively.
- Pregnant women with allergic rhinitis or atopic dermatitis exhibited worse depressive symptoms and physical component QoL scores than women without allergies.
- Severe allergic symptoms were associated with severe depressive symptoms and worsened physical component QoL scores among pregnant women.
- About 30% of women could not consult a physician regarding their symptoms and taking medicine during pregnancy.

## Introduction

Approximately half of the pregnant women have allergic diseases, such as allergic rhinitis, atopic dermatitis (AD), allergic conjunctivitis, asthma, and food allergy, in Japan. In particular, women with allergic rhi nitis (36.0%) and AD (15.7%) are common.^1^ Allergies cannot be completely cured and thereby necessitate a focus on palliative factors for their prevention or symptom control. Treatment options for nonpregnant women with allergic rhinitis or AD include prescription or over-the-counter medications. However, pregnant women with allergies face several difficulties in avoiding medications that can affect fetal development. Women who have achieved symptom control using medicines may, on discontinuing these medicines during pregnancy, experience symptom exacerbation and may need to endure allergy symptoms throughout pregnancy, thereby increasing their difficulties. During pregnancy, 26.4% of pregnant women with AD experienced exacerbations, possibly because AD is a T helper 2 cell-dominant disease that may worsen during pregnancy; however, the same study showed that 15.4% of pregnant women with AD had symptom improvement,^2^ and the reason for this discrepancy is unknown.

Pregnant women with allergies (defined as positive immunoglobulin E sensitization) are at risk of depression and lower quality of life (QoL).^3^ Moreover, pregnant women with allergic rhinitis had lower rhinosinusitis-specific QoL than those without rhinitis.^4^ One study reported the concern that pregnant women with AD have with regard to passing on the allergy to their child.^1^ However, very few studies have investigated the difficulties faced by pregnant women with allergies, their symptom management, and the association between allergies and mental health or QoL. Medical workers are generally not interested in the aforementioned aspects, as these factors may not carry a related risk of obstetric complications.

We hypothesized that the severity of allergic symptoms and the preconceptional allergy-control methods may affect the change in the status of symptoms (exacerbation or improvement) and mental health during pregnancy. This study had 2 objectives: to examine whether allergies, particularly the changes in allergy severity during pregnancy, affect the mental health and QoL of pregnant women and to investigate the relationship between changes in allergic symptoms during pregnancy and the allergy severity or symptom-control methods used preconceptionally.

## Materials and methods

### Study design

This cross-sectional study was conducted between March 18, 2020, and 26, 2020, through an online questionnaire survey, during the COVID-19 pandemic, when there was no lockdown in Japan. First, we administered a screening questionnaire about pregnancy and history of allergic disease to approximately 20,000 women monitors registered with the online-survey company (Cross Marketing Inc., Tokyo, Japan). Next, participants who met the inclusion criteria were asked to respond to the online self-administered study questionnaire. Inclusion criteria were pregnant women at the time. We targeted to collect responses from 150 women in each of the 3 groups: AD, allergic rhinitis, or no allergies. Exclusion criteria were women had allergies only during childhood. The survey ended when the target sample size was achieved. The research ethics committee of the Graduate School of Medicine, The University of Tokyo, approved (Number 2019317NI) our study protocol, which assured patient anonymity. Written informed consent was obtained from all participants before they answered the questionnaire.

### Participants

The following variables were included in the questionnaire: sex, presence or absence of pregnancy, and self-reported allergies. Questions on allergies included, “Have you had any allergies during adulthood?” Multiple answers were accepted for the following choices: allergic rhinitis, allergic conjunctivitis, AD, asthma, food allergy, allergies only in childhood, and no allergic diseases. Based on the responses to these choices, we recruited participants for the allergic rhinitis, AD, and no allergy groups (which included positive responses to the options: allergies only during childhood and none of the allergic diseases). If participants had both allergic rhinitis and AD, they were counted as the AD group because the number of participants with AD was estimated to be less than that of participants with rhinitis.

### Variables

#### Allergic symptoms: allergic rhinitis

The presence of AD or rhinitis was self-reported, and the severity of the allergic rhinitis symptoms was defined using the guidelines for nasal allergy care, which evaluates symptoms, such as sneezing, nasal discharge, and nasal congestion,^5^ and the details of responses to each question and the classification of severity are shown in Table 1. We evaluated symptoms during 2 different phases: before pregnancy and during pregnancy.

**Table 1.** Severity of rhinitis definition

Nasal congestion
	Sneeze or nasal discharge per day				
	21 times and more per day	11–20 times per day	6–10 times per day	1–5 times per day	Less than 1 time per day
Complete blockage throughout the day	Very severe	Very severe	Very severe	Very severe	Very severe
Very strong nasal obstruction with predominant mouth breathing during the day	Very severe	Severe	Severe	Severe	Severe
Strong nasal obstruction with occasional mouth breathing during the day	Very severe	Severe	Moderate	Moderate	Moderate
Nasal obstruction with no mouth breathing during the day	Very severe	Severe	Moderate	Mild	Mild
No nasal congestion	Very severe	Severe	Moderate	Mild	None

#### Allergic symptoms: atopic dermatitis

The classification of the severity of AD symptoms was defined using the Patient-Oriented Eczema Measure,^6^ which focuses on the severity of atopic eczema as experienced by the patient. Patient-Oriented Eczema Measure includes 7 items, whose total score ranges from 0 to 28. Severity is defined based on the following criteria: total score of 0–2 indicates none; 3–7, mild; 8–16, moderate; 17–24, severe; and 25–28, very severe.^7^

### Methods for controlling allergic symptoms

For both allergic rhinitis and AD, we asked participants about the methods of symptom control used before and during pregnancy as follows: visiting a clinic and consuming the prescribed medications (regularly or when necessary), purchasing over-the-counter medicines, using masks and glasses without medication, and not doing anything in particular. Participants could choose multiple options. We inquired about changes in allergic symptoms: “Did you experience any changes in your allergic symptoms during pregnancy?” The responses were no change, exacerbation, and improved symptoms when compared with the preconceptional state.

### Mental health and QoL

Mental health was evaluated using the Kessler Psychological Distress Scale (K6),^8^ which includes 6 items with a total score from 0 to 24. Severe depressive symptoms and depressive symptoms were defined based on a total score ≥13 and ≥5, respectively.

Health-related QoL was measured using the Medical Outcomes Survey Short Form-8 questionnaire,^9^ which includes 8 items; the scores were presented as the national standard score of 50 points. The Short Form-8 has 2 summary scores: namely, physical component summary and mental component summary; higher scores indicate a higher quality of health status.

### Demographics

Data on the following demographic variables were collected: age, gestational age, educational level, number of children, history of psychiatric consultations, and allergen characteristics.

### Statistical methods

First, we examined whether allergies, particularly the severity and changes in allergy symptoms during pregnancy, affect the mental health and QoL of pregnant women. We investigated whether the pregnancy-induced effect persists after adjusting for a history of psychiatric consultation. Second, we investigated the relationship between changes in allergy symptoms during pregnancy and the allergy severity or control methods used before pregnancy using a multiple logistic regression model. All statistical analyses were performed using SPSS, version 22.0 (IBM Corp., Armonk, NY). All *P* values were 2-sided, and a *P* < .05 was considered statistically significant.

## Results

The questionnaire was answered by 434 pregnant women and, after excluding participants with <8 weeks of gestation, the data of 411 participants were analyzed. Of the 411 women, 144 had no allergies, 210 had allergic rhinitis, 119 had AD, and 62 had both allergic rhinitis and AD. Table 2 shows the background characteristics of the participants.

**Table 2.** Characteristics of all study participants

Characteristics	No allergies (*n* = 144)	With allergic rhinitis or atopic dermatology (*n* = 267)	*P*
Age	31.4 ± 4.4	30.7 ± 4.6	.149
Gestational age (wk)	28.2 ± 7.6	27.0 ± 8.0	.138
Work			
Housewife, maternity retirement, or leave	90 (62.5%)	163 (61.0%)	.156
Workplace where you can take a break	45 (31.3%)	72 (27.0%)	
Workplace where you cannot take a break	8 (5.6%)	23 (8.6%)	
Freelance and self-employed	1 (0.7%)	9 (3.4%)	
Primipara	71 (49.3%)	137 (51.3%)	.367
History of psychiatric consultations	16 (11.1%)	43 (16.1%)	.168
K6			
Score	5.5 ± 5.1	5.8 ± 5.9	.593
Depression (score 5 and more)	75 (52.1%)	127 (47.6%)	.382
Severe depression (score 13 and more)	9 (6.3%)	36 (13.5%)	.025
SF-8			
PCS	45.4 ± 7.5	44.9 ± 6.9	.500
MCS	48.8 ± 6.7	47.6 ± 7.4	.086
Pregnancy and Medication Counseling Center			
I did not know it	137 (95/1%)	238 (89.1%)	.040
I saw a doctor	2 (1.4%)	7 (2.6%)	.415

MCS, mental component summary; PCS, physical component summary; SD, standard deviation; SF-8, Short Form-8; wk, weeks.

Values are expressed as *n* (%) and mean ± SD.

First, we evaluated allergic rhinitis (*n* = 210): 82 (39.0%) and 84 (40.0%) women had severe or very severe symptoms before and during pregnancy, respectively. Regardless of their symptoms, 36 (17.1%), 53 (25.2%), and 121 (57.6%) participants experienced exacerbation, improved symptoms, or no change, respectively. Second, we evaluated AD (*n* = 119): 14 (11.8%) and 22 (18.5%) women had severe or very severe symptoms before and during pregnancy, respectively. Regardless of their symptoms, 36 (30.3%), 13 (10.9%), and 70 (58.8%) participants experienced exacerbation, improved symptoms, or no change, respectively.

### Relationship between allergies and mental health

**Fig. 1.**
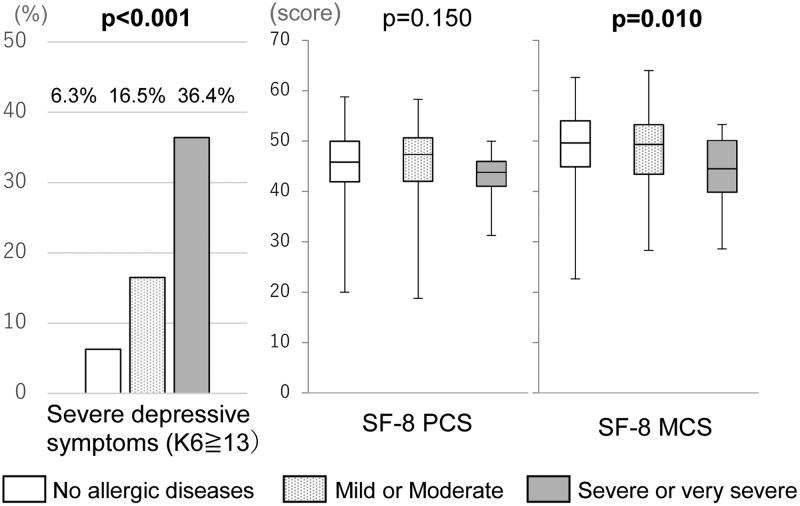
Relationship between AD symptoms during pregnancy and mental health. AD, atopic dermatitis; MCS, mental component summary; PCS, physical component summary; SF-8, Short Form-8.

**Fig. 2.**
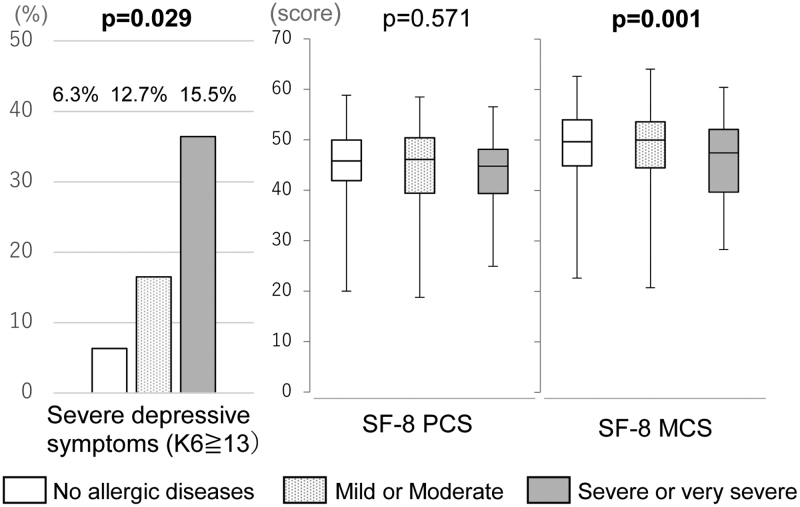
Relationship between allergic rhinitis symptoms during pregnancy and mental health. MCS, mental component summary; PCS, physical component summary; SF-8, Short Form-8.

We examined the relationship of allergy symptoms with mental health and QoL. More severe depressive symptoms and a worsened physical component of QoL were observed in women with symptoms of both AD and allergic rhinitis than in women without allergies (Figs. 1 and 2). Women with severe symptoms of AD experienced significantly severe depressive symptoms and lower QoL, even after adjusting for a history of psychiatric consultations (Supplementary Table S1, http://links.lww.com/IJWD/A0). Severe symptoms of rhinitis resulted in a worsened physical component of QoL, even after adjusting for a history of psychiatric consultations. There was no relationship between change in symptoms during pregnancy and mental health (Table 3).

**Table 3.** Relationship between change of symptoms during pregnancy and mental health

Mental health	Improved symptoms	No change	Exacerbation	*P*
Atopic dermatitis	*n* = 13 (10.9%)	*n* = 70 (58.8%)	*n* = 36 (30.3%)	
Severe depression	4 (30.8%)	14 (20.0%)	6 (16.7%)	.554
SF-8				
PCS	45.7 ± 8.5	46.1 ± 6.6	43.2 ± 7.2	.126
MCS	49.1 ± 5.8	46.9 ± 7.1	45.9 ± 8.0	.388
History of psychiatric consultations	3 (23.1%)	13 (18.6%)	9 (25.0%)	.730
Allergic rhinitis	*n* = 53 (25.2%)	*n* = 121 (57.6%)	*n* = 36 (17.1%)	
Severe depression	8 (15.1%)	16 (13.2%)	5 (13.9%)	.947
SF-8				
PCS	44.2 ± 6.4	44.7 ± 6.9	44.6 ± 9.1	.906
MCS	44.6 ± 7.2	46.9 ± 7.4	48.3 ± 7.7	.100
History of psychiatric consultations	7 (13.2%)	21 (17.4%)	8 (22.2%)	.539

MCS, mental component summary; PCS, physical component summary; SD, standard deviation; SF-8, Short Form-8.

Values are expressed as *n* (%) and mean ± SD.

### Relationship between exacerbation of allergic symptoms during pregnancy and severity or control methods used preconceptionally

We investigated the factors influencing exacerbations of allergic symptoms during pregnancy. With regard to AD, bivariate analysis showed severe symptoms before pregnancy. The job situation of participants and methods for achieving AD symptom control during pregnancy resulted in changes in symptoms, whereas the symptoms were exacerbated during pregnancy (Supplementary Table S2, http://links.lww.com/IJWD/A0). After adjusting for each factor, severe prepregnancy symptoms, job situation of participants (stay-at-home roles, such as housewife, on maternity leave, retired from work during childbirth), and coping with symptoms of AD during pregnancy were significantly associated with exacerbation of AD (Table 4). There was no relationship between change in symptoms and the methods for controlling AD symptoms preconceptionally. Notably, 11 women (30.6%) with exacerbation of AD during pregnancy did not consult a practitioner and endured their exacerbation.

**Table 4.** Risk factors significantly associated with experience exacerbation of AD during pregnancy (*n* = 119)^a^

Variables	COR (95% CI)	*P*	AOR^b^ (95% CI)	*P*
Severity of AD before pregnancy				
Mild or moderate	Ref		Ref	
Severe and very severe	3.67 (1.17, 11.51)	.026	4.57 (1.23, 17.01)	.023
Job situation				
Workplace where you can take a break/freelance and self-employed	Ref		Ref	
Housewife, maternity retirement, maternity leave	3.56 (1.31, 9.66)	.013	4.41 (1.42, 13.66)	.010
Workplace where you “cannot” take a break	4.17 (0.99, 17.55)	.052	3.60 (0.79, 16.36)	.098
Coping with symptoms of AD during pregnancy				
Coping without medication	Ref		Ref	
Visiting the hospital and coping with medication	6.24 (2.08, 18.77)	.001	5.81 (1.83, 18.47)	.003
Enduring the symptoms	4.5 (1.37, 14.86)	.013	5.88 (1.63, 21.20)	.007

95% CI, 95% confidence interval; AD, atopic dermatitis; AOR, adjusted odds ratio; COR, crude odds ratio; Ref, reference.

^a^ It was assessed using logistic regression analysis (worsening symptoms *n* = 36, improved symptoms or no change *n* = 83).

^b^ AOR (adjusted for variables in table).

In patients with allergic rhinitis, bivariate analysis revealed that severe symptoms before pregnancy, gestational age, presence of asthma, and presence of rhinitis symptoms from December to February were significantly associated with symptom exacerbation during pregnancy (Supplementary Table S3, http://links.lww.com/IJWD/A0). After adjusting for each factor, the winter season (between December and February) and the second trimester of gestation were significantly associated with the exacerbation of rhinitis (Table 5).

**Table 5.** Risk factors significantly associated with experience exacerbation of rhinitis during pregnancy (*n* = 210)^a^

Variables	COR (95% CI)	*P*	AOR^b^ (95% CI)	*P*
Severity of rhinitis before pregnancy				
Mild or moderate	Ref		Ref	
Severe and very severe	0.46 (0.21, 1.04)	.062	0.43 (0.18, 1.01)	.053
Have asthma: yes	2.82 (1.10, 7.22)	.030	2.59 (0.95, 7.07)	.063
Have rhinitis symptoms in winter (between December and February)	2.61 (1.16, 5.88)	.020	2.75 (1.18, 6.43)	.020
Gestational age				
First trimester	3.13 (0.95, 10.34)	.062	3.24 (0.93, 11.31)	.065
Second trimester	3.62 (1.60, 8.22)	.002	3.26 (1.40, 7.59)	.006
Third trimester	Ref		Ref	

95% CI, 95% confidence interval; AOR, adjusted odds ratio; COR, crude odds ratio; Ref, reference.

^a^It was assessed using logistic regression analysis (worsening symptoms *n* = 36, or no change *n* = 174).

^b^AOR (adjusted for variables in table).

## Discussion

Compared with pregnant women without allergies, those with allergies have significantly worsening mental health, especially severe depressive symptoms and a lower psychological aspect of QoL. We found that 30% and 17% of women with AD and allergic rhinitis experienced exacerbations of allergic symptoms during pregnancy. A significant association was observed among severe prepregnancy symptoms, job situation, coping with symptoms of AD during pregnancy, and exacerbation of AD. The timing of symptoms and the second trimester of gestation were significantly associated with symptom exacerbation of rhinitis.

### Participants

The participants of the present study had higher rates of depressive symptoms (total population: depressive symptoms 49.1%, severe depressive symptoms 10.9%; only women without allergic diseases: depressive symptoms 52.1%, severe depressive symptoms 6.3%) than those of the study by Yamamoto-Hanada et al.,^3^ which included a larger study sample of pregnant women in Japan (depression 31.9%, severe depression 3.5%). This finding is attributable to several reasons. First, this study specifically recruited people with allergies who tend to have a higher K6 score. The rate of depressive symptoms in pregnant women with allergic rhinitis remains high, reaffirming the importance of supporting the mental health of women with allergies during pregnancy. Second, the survey may have been biased toward individuals with mental health problems because it was a web-based survey. Finally, the survey was conducted amid the COVID-19 pandemic, which has, in general, caused anxiety among individuals, especially pregnant women.^10^ However, because all women were equally under the influence of COVID-19–related anxiety, we consider the finding that allergy affects psychological aspects to be reliable.

### Effect of allergies on the mental health and QoL of pregnant women

Compared with participants without allergies, participants with allergies have significantly worse mental health, a lower mental QoL, and a higher K6 score, especially those who are currently experiencing severe and very severe symptoms. This result did not change after adjusting for a history of psychiatric consultations. However, there was no association between the exacerbation of symptoms during pregnancy and mental health.

In the general adult population, atopic symptoms and depression are linked.^11^ Depressive symptoms are more likely to increase during pregnancy and could affect parenting as well as cause postpartum depression and bonding disorder. Currently, the association between AD and/or rhinitis and pregnancy has not received sufficient attention because it does not affect obstetric outcomes. However, as it affects mental health during pregnancy, there is a need for a multidisciplinary collaboration between allergists and obstetricians or midwives to ensure that pregnant women consult experts to reduce AD symptoms. Depression and anxiety during pregnancy increase the risk of the fetus developing AD postnatally through epigenetic changes in the placenta.^12,13^ Children born to mothers with AD are at high risk of developing AD; however, a low level of distress may reduce the risk of developing the disease. Our findings of the relationship between atopic symptoms and mental health during pregnancy suggest that controlling and reducing symptoms during pregnancy is beneficial for the fetus.

The absence of an association between symptom exacerbation and mental health may be because the exacerbation was not strongly associated with symptom severity during pregnancy. People who experience strong symptoms before pregnancy are unlikely to feel that the symptoms have worsened. Therefore, it would be important to address the intensity of the current symptoms and not the changes that have occurred since pregnancy.

### Relationship between changes in allergic symptoms during pregnancy and allergy severity or control methods used preconceptionally

Concerning AD, high-risk factors that were significantly associated with symptomatic changes included the preconceptional allergy severity, which was either severe or very severe; job situation, which included either housewife or maternity retirement or maternity leave; and coping style for AD symptoms, which included visiting the hospital to obtain a prescription or enduring the symptoms.

Before the survey, we assumed that a home-bound, stress-free resting period would improve atopic symptoms. However, the option “Workplace where you can take a break/freelance and self-employed” was associated with a lower risk of exacerbations than other options, whereas “stay home” and “workplace where they cannot take a break” were associated with a significantly high risk of exacerbations. We hypothesized 2 explanations. First, staying at home was stressful for pregnant women and a workplace where they could take breaks was preferred because appropriate social activity reduces stress more than staying at home for longer periods. Exacerbation of AD was related to stress and disturbed mental health.^14^ Continued engagement in social activities during pregnancy for stress reduction may be important for controlling atopy-related symptoms. Second, household work, especially kitchen work, may exacerbate symptoms, such as those of contact dermatitis. In addition, house dust mites may have exacerbated allergic symptoms in some pregnant participants who spent more time at home than they had spent before conceiving.

Patients who visited the hospital after their symptoms exacerbated did not face any problems; however, those who endured symptom exacerbation faced added complications. Surprisingly, approximately one-fourth of the participants endured exacerbation of symptoms. Pregnant women were hesitant to take medications owing to the fear of obstetric complications; however, certain medications are safe to use. Our study revealed that pregnant women were not aware of the Pregnancy and Medication Counseling Center,^15^ which was established to respond to inquiries from pregnant women about medication-related issues. It is necessary to raise awareness among pregnant women regarding the adverse effects of enduring exacerbation instead of seeking help and/or consultation.

Among patients with rhinitis, symptoms during December–February and during the second trimester of pregnancy were associated with symptom exacerbation. Interestingly, the second trimester was a risk factor for exacerbations, whereas symptom improvement was observed in the third trimester. These findings were similar to those reported in a previous study about asthma that reported more moderate/severe symptoms in the second trimester than in the third trimester.^16^ The cause of this difference between the 2 trimesters was unknown, although it could possibly be attributable to the fact that pregnant women establish their own coping strategies during pregnancy and adapt to them. In contrast, the symptoms in winter may have shown associations because they occurred in the period immediately before the study period (March), facilitating more accurate recall by the participants.

The reason for the discrepancy in the results between AD and rhinitis might be that most participants with rhinitis had milder symptoms than those with AD. We included “hay fever” for rhinitis, which may have led to the inclusion of individuals who were not diagnosed, whereas the category of AD may have included those who were diagnosed with severe symptoms, which possibly affected the results.

Our study has some limitations. First, the cross-sectional design could not facilitate an investigation of the changes in the symptoms of each participant. Women in the third trimester had milder symptoms from before pregnancy than those in the second trimester. Future studies must carefully monitor the changes in each woman’s symptoms during different periods of pregnancy. Second, both symptoms and diagnoses were self-reported, and the presence or absence of allergic disease was not confirmed by a specialist; therefore, the number of allergic patients may have been overestimated. Finally, recall bias would be present since the pregnant participants answered the questionnaires by recalling their prepregnancy symptoms.

Despite these limitations, this study found an association between the severity of allergic symptoms and mental health in pregnant women. Furthermore, we evaluated the proportion of women with exacerbations during pregnancy and the factors that affected those symptoms. These results reveal that women with allergies face multiple challenges and require support during various phases of pregnancy, and we hope to expand our research on how to support these individuals in the future.

## Conclusion

Perinatal care professionals or allergists should consider the psychological aspects of pregnant women with AD or allergic rhinitis. Particular attention should be focused not only on the kind of disease but also on the symptom severity; for example, if one experiences severe AD, additional attention should be paid to depressive symptoms during pregnancy. Additionally, even among women with controlled symptoms, 30.3% and 17.1% of participants who had AD and allergic rhinitis, respectively, experienced exacerbations during pregnancy and felt helpless, as they could not consult a practitioner regarding their symptoms. Therefore, it is crucial for an allergist, obstetrician, and/or midwife to collaborate and provide appropriate information and support to pregnant women with allergies.

## Author contributions

All authors designed the study, performed interpretation of the results and approved the final manuscript. K.Y. performed the statistical analysis and wrote the manuscript draft.

## Conflicts of interest

None.

## Funding

This study was supported by the program to support faculties with their restart-up research at The University of Tokyo in 2019.

## Study approval

The author(s) confirm that any aspect of the work covered in this manuscript that has involved human patients has been conducted with the ethical approval of all relevant bodies.

## Patient consent

Informed written consent was received from all patients and confirmed to the journal prepublication.

## Supplementary materials

Supplementary material associated with this article can be found at http://links.lww.com/IJWD/A0.

## Supplementary Material
