# Supplementary material for: Exacerbation and severity of allergic symptoms during pregnancy and their impact on mental health
Source: Int J Womens Dermatol. 2022 Mar 22;8(1):e002. doi: 10.1097/JW9.0000000000000002 (PMC9112393; doi:10.1097/JW9.0000000000000002)
Supplement: Supplementary file 1 [file jw9-8-e002-s001.pdf]

Supplementary Table 1 Impact of AD symptoms on severe depression

	COR	95%CI	*p–value*	AOR	95%CI	*p–value*
**Severity of AD**						
No allergy	Ref			Ref		
mild or moderate	1.94	1.01 – 3.75	0.026	2.28	1.12 – 4.65	0.023
severe and very severe	5.44	2.14 – 13.81	<0.001	6.81	2.50 – 18.55	<0.001
**History of psychiatric consultations**	3.66	1.83 – 7.33	<0.001	3.24	1.57 – 6.68	0.001

Supplementary Table 2 Characteristics of change of AD symptoms during pregnancy

	Improved symptoms or no change n=83	Exacerbation of symptoms n=36	*p–value*
**Severity of AD before pregnancy**			0.020
mild or moderate	77 (92.8%)	28 (77.8%)	
severe and very severe	6 (7.2%)	8 (22.2%)	
**Age**	30.7 ± 4.6	30.3 ± 4.6	0.661
Gestational age (weeks)	24.9 ± 8.2	26.0 ± 7.8	0.491
**Work**			0.026
Housewife, maternity retirement or leave	41 (49.4%)	25 (69.4%)	
Workplace where you can take a break	35 (42.2%)	6 (16.7%)	
Workplace where you cannot take a break	7 (8.4%)	5 (13.9%)	
**History of psychiatric consultations**	16 (19.3%)	9 (25.0%)	0.481
**Allergen**			
pollen	61 (73.5%)	23 (63.9%)	0.291
mildew	24 (28.9%)	10 (27.8%)	0.900
dust mites and house dust	56 (67.5%)	20 (55.6%)	0.214
Animal Hair and Feathers	29 (34.9%)	12 (33.3%)	0.865
foods	5 (6.0%)	3 (8.3%)	0.644
medicine	4 (4.8%)	2 (5.6%)	0.866
chemicals	13 (15.7%)	6 (16.7%)	0.891
**Coping with symptoms of AD before pregnancy**			0.460
Take care without medicine	40 (48.2%)	15 (41.7%)	
Going to hospital to take Medicine	47 (56.6%)	23 (63.9%)	
**Coping with symptoms of AD during pregnancy**			0.010
Take care without medicine	35 (42.2%)	5 (13.9%)	
Going to hospital to take Medicine	29 (34.9%)	20 (55.6%)	
Enduring the symptoms.	19 (22.9%)	11 (30.6%)	

Supplementary Table 3 Characteristics of change of rhinitis symptoms during pregnancy

	Improved symptoms or no change n=174	Exacerbation of symptoms n=36	*p–value*
**Severity of rhinitis before pregnancy**			0.058
mild or moderate	101 (58.0%)	27 (75.0%)	
severe and very severe	73 (42.0%)	9 (25.0%)	
**Age**	31.1 ± 4.6	30.0 ± 5.1	0.212
**Gestational age (weeks)**	28.3 ± 7.9	23.5 ± 7.1	0.001
**Work**			0.532
Housewife, maternity retirement or leave	104 (59.8%)	25 (69.4%)	
Workplace where you can take a break	54 (31.0%)	8 (22.2%)	
Workplace where you cannot take a break	16 (9.2%)	3 (8.3%)	
**History of psychiatric consultations**	28 (16.1%)	8 (22.2%)	0.374
**Allergen**			
pollen	158 (90.8%)	30 (83.3%)	0.183
mildew	28 (16.1%)	9 (25.0%)	0.202
dust mites and house dust	97 (55.7%)	19 (52.8%)	0.744
Animal Hair and Feathers	48 (27.6%)	9 (25.0%)	0.751
foods	8 (4.6%)	4 (11.1%)	0.125
medicine	3 (1.7%)	2 (5.6%)	0.170
chemicals	17 (9.8%)	5 (13.9%)	0.463
**Season with rhinitis symptoms**			
All year round (n=35)	28 (16.1%)	7 (19.4%)	0.623
Spring (March-May, n=151)	127 (73.0%)	24 (66.7%)	0.442
Summer (June-Aug, n=14)	11 (6.3%)	3 (8.3%)	0.660
Autumn (Sep-Nov, n=39)	32 (18.4%)	7 (19.4%)	0.882
Winter (Dec-Feb, n=120)	93 (53.4%)	27 (75.0%)	0.017
**Coping with symptoms of rhinitis before pregnancy**			0.248
Take care without medicine	82 (47.1%)	20 (55.6%)	
over-the-counter drug	28 (16.1%)	2 (5.6%)	
Going to hospital to take Medicine	64 (36.8%)	14 (38.9%)	
**Coping with symptoms of rhinitis during pregnancy**			0.144
Take care without medicine	85 (48.9%)	10 (27.8%)	
over-the-counter drug	4 (2.3%)	1 (2/8%)	
Going to hospital to take Medicine	39 (22.4%)	12 (33/3%)	
Enduring the symptoms.	46 (26.4%)	13 (36.1%)	
